# SGOL2 promotes prostate cancer progression by inhibiting RAB1A ubiquitination

**DOI:** 10.18632/aging.204443

**Published:** 2022-12-23

**Authors:** Tingting Lv, Dongwei He, Xiaokuan Zhang, Xiaojin Guo, Zijie Li, Aili Zhang, Bo Fan, Zhiyu Wang

**Affiliations:** 1 Department of Immuno-Oncology, The Fourth Hospital of Hebei Medical University, Shijiazhuang 050011, Hebei, P.R. China; 2 Department of Urinary Surgery, The Fourth Hospital of Hebei Medical University, Shijiazhuang 050011, Hebei, P.R. China

**Keywords:** prostate cancer, SGOL2, RAB1A, ubiquitination, tumor microenvironment

## Abstract

Prostate cancer is the most prevalent genitourinary malignant cancer in men worldwide. Patients with prostate cancer who progress to castration-resistant prostate cancer (CRPC) or metastatic CRPC have significantly poorer survival. Advanced prostate cancer is a clinical challenge due to the lack of effective treatment strategies. In the field of oncology, SGOL2 was an emerging and differentially expressed molecule, which enhanced the proliferation of cell populations *in vitro* in our studies. Mass spectrum and Co-IP validated the interaction of SGOL2 and RAB1A in a protein-protein manner. We further investigated the role of SGOL2 in the regulatory mechanism of RAB1A in prostate cancer cell lines. Furthermore, SGOL2 regulated RAB1A expression by inhibiting its ubiquitination. Rescue Experiments demonstrated that SGOL2 promoted prostate cancer cell proliferation and migration by upregulating RAB1A expression. Finally, we found that SGOL2 and RAB1A may regulate the tumor microenvironment (TME) in prostate cancer. In conclusion, our findings concluded that SGOL2 stabilized RAB1A expression to promote prostate cancer development. Both of them were of great importance in TME modulation.

## INTRODUCTION

Prostate cancer has been newly demonstrated to have the third highest incidence of new cases among 36 cancers, behind breast cancer and lung cancer [1]. As the second most common cancer in old males around the world, therapeutic strategies remain fixed, such as radical prostatectomy and radiotherapy for localized prostate cancer and androgen deprivation treatment for advanced and metastatic forms of prostate cancer [2]. Though immunotherapy and targeted therapies have been applied in tumor treatment, the prognostic improvement for advanced prostate cancer is still slight. The pathogenesis of prostate cancer, especially progressing to metastatic castration-resistant prostate cancer (mCRPC), is a sophisticated process. Due to a boom in the field of genetics and bioinformatics, several large-scale genomic studies indicated the existence of mutations, rearrangements, gene fusion, and DNA copy number changes in primary and advanced prostate cancer [3, 4]. Therefore, there is an urgent need to link genetic abnormalities with strategies administered in personalized treatment for prostate cancer patients.

Shugoshin 2 (SGO2, also known as SGOL2), which is tightly involved in the cell cycle process, has been reported to protect cohesion, sustain the linkage of chromosomes and regulate kinetochore-microtubule attachment in meiosis and mitosis [5–10]. Additionally, SGOL2 modulates the function of the subtelomere and improves HSP70 expression during heat shock to support cell survival and protein homeostasis [11, 12]. A growing body of evidence indicates that SGOL2 is a novel molecule with profound significance in cancer and related fields. Up to now, what is clear is that SGOL2 has been reported as a differentially expressed gene in various types of cancer, including glioma, hepatocellular cancer, and endometrial cancer [13–15]. Previous studies had demonstrated that SGOL2 had a protein-protein interaction with BRCA1, whose variants were regarded as biomarkers to predict the survival prognosis of prostate cancer patients [16, 17]. However, the role of SGOL2 in prostate cancer development and progression remains incompletely understood.

Ubiquitin is an 8.5-kDa, 76 amino acid polypeptide, which was first described in the structure of chromosomal conjugate-protein A24 as a post-translational modifier [18, 19]. The process of ubiquitination is a sequential enzymatic cascade, including three enzymatic steps: a ubiquitin-activating enzyme (E1), a ubiquitin-conjugating enzyme (E2), and a ubiquitin ligase (E3) [20]. Ubiquitination is a dynamically multifaceted post-translational modification involved in many physiological activities, such as cell cycle, autography, tumorigenesis, etc. A lot of ubiquitination modifications were responsible for the development and progression of prostate cancer. Previous studies indicated that E3 ligase MDM2, STUB1, and DCAF11 were involved in androgen receptor degradation and induced androgen receptor targeted therapy resistance [21–23]. TRAF4, an E3 ubiquitin ligase, could mediate TrkA ubiquitination to active TrkA signaling pathway and thus modulate prostate cancer progression with the presence of NGF [24]. All in all, exploration of ubiquitination modifications in prostate cancer is of great significance to develop efficient therapeutic targets.

In our study, SGOL2 was highly expressed in prostate cancer compared to adjacent tissue and confirmed as a pro-tumor regulator. Further exploration of the downstream mechanism elucidated that SGOL2 positively regulated RAB1A expression, a member of the GTPase family, by inhibiting its ubiquitination modification. Finally, based on our bioinformatic findings, we conjectured that SGOL2 and RAB1A both contributed to tumor microenvironment (TME) modulation in prostate cancer.

## RESULTS

### SGOL2 was overexpressed in prostate cancer and strongly associated with cancer development

**Figure 1:**
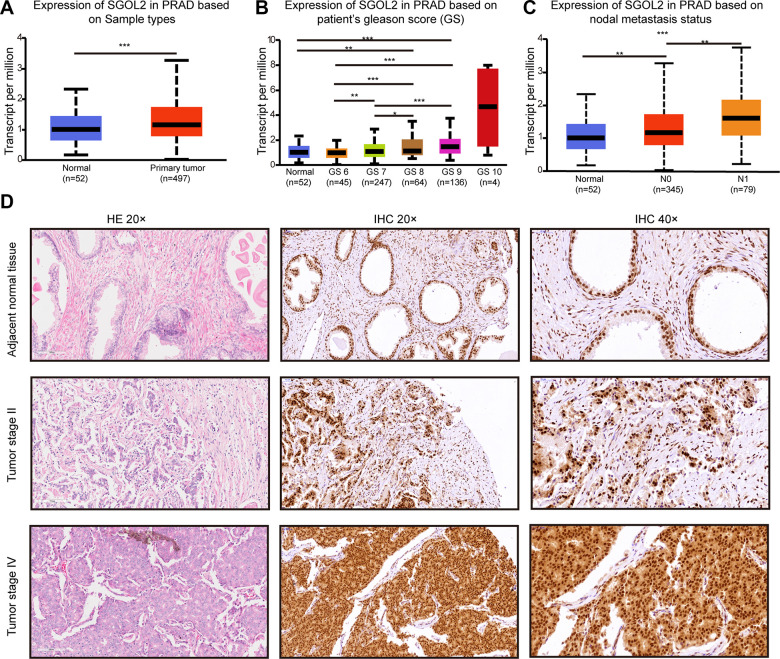
**The levels of SGOL2 expression upregulated in prostate cancer.** (**A**) TCGA analysis showed SGOL2 expression in prostate cancer tissue and adjacent normal tissue. (**B**) The relationship between SGOL2 expression and the Gleason score of patients. (**C**) Correlation between SGOL2 expression and nodal metastasis status. (**D**) Representative IHC images for SGOL2 expression level in prostate cancer tissue microarrays. *, P<0.05; **, P<0.01; ***, P<0.001.

Considering the potential role of SGOL2 in tumorigenesis and cancer development, we first detected SGOL2 expression in prostate cancer in UALCAN [25], which revealed a significantly higher SGOL2 expression in prostate cancer tissues than in adjacent normal tissues. Moreover, SGOL2 expression tightly correlated with clinical stage and lymphatic metastasis (P<0.001) (Figure 1A–1C). We performed immunohistochemistry (IHC) to detect SGOL2 expression among 91 prostate cancer patients, which indicated that higher-grade prostate cancer expressed higher SGOL2 expression (Figure 1D). Furthermore, we evaluated the potential correlation between SGOL2 protein expression and clinicopathological characteristics and found that SGOL2 expression positively correlated with Gleason Score (r=0.213, P=0.043), pathological grade (r=0.285, P=0.007), lymphatic metastasis (r=0.341, P<0.001) and clinical stage (r=0.409, P<0.001). It demonstrated that the level of SGOL2 had no significant effect on age (P=0.180) and primary tumor volume (P=0.268) (Table 1 and Supplementary Table 2).

**Table 1:** Relationship between SGOL2 expression and tumor characteristics in patients with prostate cancer.

**Features**	**Number of patients**	**SGOL2 expression**		**p value**
		**Low**	**High**	
All patients	91	44	47	
Age (years)				0.180
≤71	43	24	19	
>71	48	20	28	
Gleason Score				0.043*
≤7	50	29	21	
>7	41	15	26	
Grade				0.007**
1	6	5	1	
2	24	17	7	
3	20	7	13	
4	18	6	12	
5	23	9	4	
T Infiltrate				0.268
T2	12	10	2	
T3	57	25	32	
T4	16	9	7	
Lymphatic metastasis (N)				0.001**
N0	69	40	29	
N1	22	4	18	
Stage				0.000***
2	10	9	1	
3	59	31	28	
4	22	4	18	

*, P<0.05; **, P<0.01; ***, P<0.001.

### The shSGOL2 model was constructed and evaluated in prostate cancer cell lines

**Figure 2:**
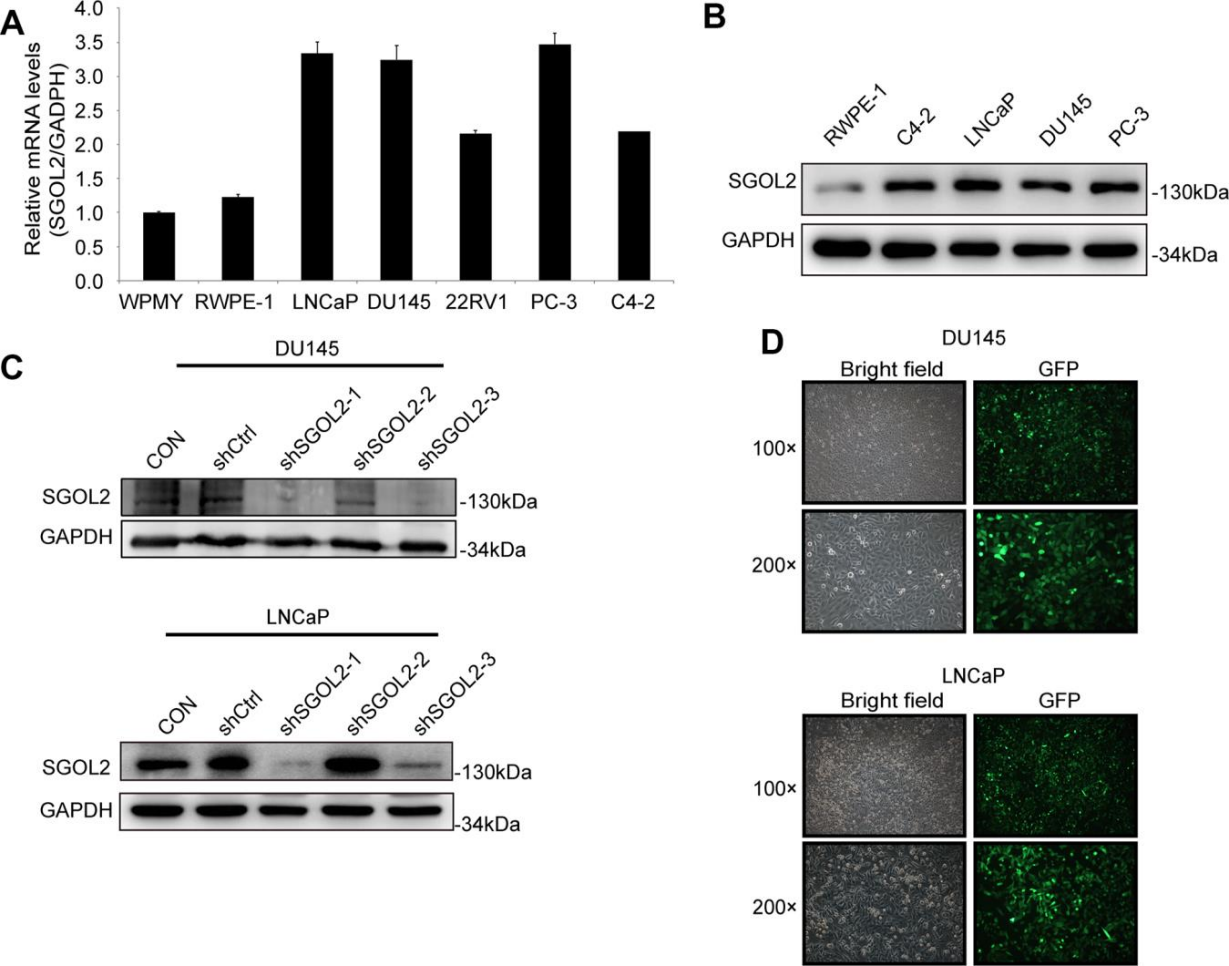
**The construction of the shSGOL2 model was evaluated in prostate cancer cell lines.** (**A**) Expression level of SGOL2 was detected in normal prostate cell lines and prostate cancer cell lines by qRT-PCR. (**B**) Efficiency of SGOL2 knockdown was accessed by WB in different cell lines. (**C**) Efficiency of SGOL2 knockdown was examined by WB in DU145 and LNCaP. (**D**) The fluorescence intensity and morphology of infected cells were observed in DU145 and LNCaP.

To select appropriate cell lines, we detected the SGOL2 expression through quantitative real-time PCR (qRT-PCR) in LNCaP, DU145, PC-3, C4-2, 22RV1 prostate cancer cell lines compared to RWPE-1 and WPMY. We also detected SGOL2 expression through Western blot (WB) in LNCaP, DU145, PC-3, C4-2, and RWPE-1. We found higher SGOL2 expression in prostate cancer cell lines at both the mRNA and protein dimensions (Figure 2A, 2B). And thus, LNCaP and DU145 were used as cell models to carry out a subsequent series of assays to assess proliferative and migratory abilities of prostate cancer cells. We downregulated SGOL2 expression of DU145 and LNCaP using shSGOL2 lentivirus, evaluated the transfection efficiency by WB, and chose shSGOL2-1 and shSGOL2-3 to construct appropriate prostate cell lines for further analysis (Figure 2C, 2D).

### SGOL2 knockdown inhibited prostate cancer proliferation and migration *in vitro*

**Figure 3:**
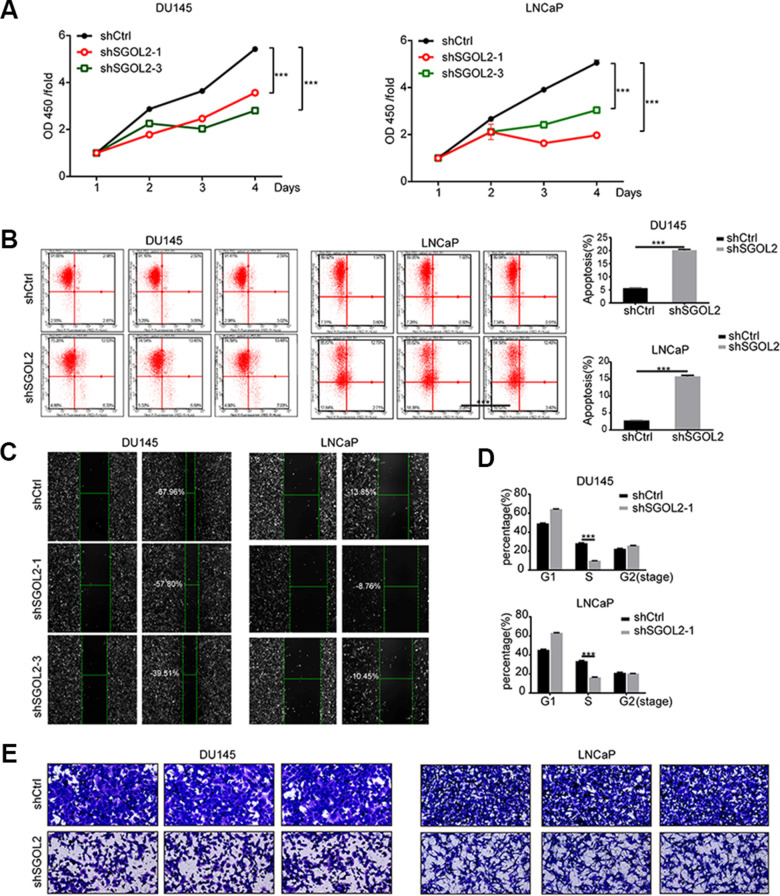
**SGOL2 deletion inhibited prostate cancer proliferation and migration.** (**A**) Cell proliferation was measured by CCK8 assay at 24h, 48h, 72h, and 96h after transfecting with shSGOL2-1, shSGOL2-3 and the control group for 48h. (**B**) Apoptosis of prostate cancer was performed after knocking down SGOL2 (shRNA-3) for 48h. (**C**) Wound-healing assay presented the metastatic ability in shSGOL2-3 and shCtrl groups. (**D**) Cell cycle assay revealed the proportion of cells in different cell phases after transfection. (**E**) Transwell assay presented the migratory cells between shSGOL2-3 and the control group. *, P<0.05; **, P<001; ***, P<0.001.

Then, we next assessed and examined the proliferation and migration *in vitro* by depleting SGOL2 expression. After infection, the CCK8 assays revealed that SGOL2 knockdown slowed down cell proliferation (P<0.001) (Figure 3A). Comparable to primary tumor cell lines, SGOL2 depletion *in vitro* led to a decrease in the rate of cell survival (P<0.001) (Figure 3B). The results of the wound-healing assay indicated a significant reduction in migration of prostate cancer cell lines by SGOL2 knockdown (Figure 3C). Consistently, ectopic suppression of SGOL2 arrested prostate cancer cells in the S phase (P<0.001) and may delay the cell cycle process (Figure 3D). Transwell assays presented that the cell migration was inhibited when SGOL2 was knocked down (P<0.001) (Figure 3E). In summary, these loss-of-function results illustrated that SGOL2 promoted tumor proliferation and metastasis *in vitro*.

### SGOL2 stabilized RAB1A expression through decreasing RAB1A ubiquitination

**Figure 4:**
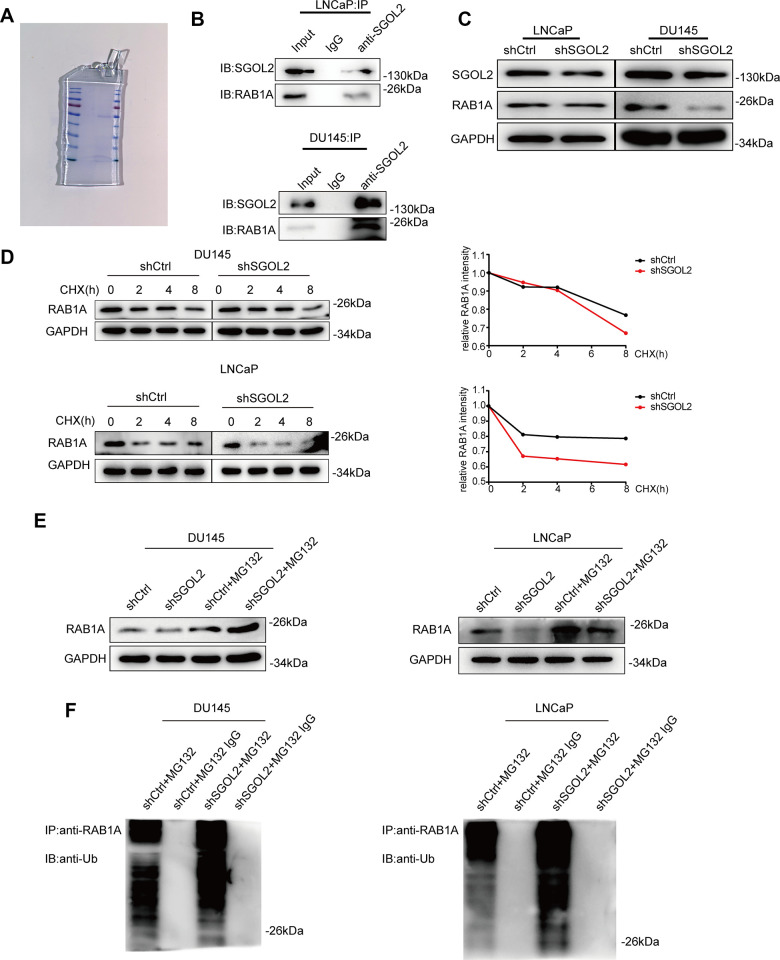
**SGOL2 stabilized RAB1A via a ubiquitination-dependent pathway.** (**A**) SDS-PAGE of the control group (left) and shSGOL2 group (right) was stained by Coomassie Blue Staining Solution. (**B**) Cell lysates were subjected to Co-IP with specific antibodies to examine the interaction between SGOL2 and RAB1A. (**C**) Detect RAB1A expression as in A in DU145 and LNCaP. (**D**) At 48h post-infection, WB analysis of RAB1A expression in shSGOL2 cell lines treated with CHX for 0, 2, 4, 8h compared to the control group. (**E**) Treated with protease inhibitor MG132 for 8h, cells were lysed and subjected to WB for RAB1A expression. (**F**) WB analysis for IPs performed with anti-ubiquitin antibody to detect SGOL2-mediated RAB1A ubiquitination.

We next set out to address the potential factors by which SGOL2 regulated the growth of prostate cancer *in vitro* by performing mass spectrometry (Figure 4A and Supplementary Figure 1A) to indicate the differentially expressed proteins upon SGOL2 knockdown. GEPIA 2.0 represented a moderate correlation between SGOL2 and RAB1A in prostate cancer, consistent with mass spectrometry prediction (Supplementary Figure 1C). Moreover, WB analysis following co-immunoprecipitation (Co-IP) also identified the relationship between the two proteins in DU145 and LNCaP (Figure 4B and Supplementary Figure 1B). In accordance with evidence from these existing results, we speculated that SGOL2 might regulate the expression of RAB1A. Subsequently, SGOL2 knockdown with RAB1A downregulation invoked the possibility that SGOL2 could contribute to RAB1A expression (Figure 4C). We further explored the underlying mechanism involved in the regulation of RAB1A. It has been widely recognized that ubiquitination, which acts as a widespread posttranslational modification, could modulate many intracellular events by regulating the activity of functional proteins in spatial and temporal dimensions [26]. The prior addition of cycloheximide (CHX) *in vitro* inhibits protein synthesis for various periods of time. Reduction in RAB1A expression in shSGOL2 models compared to the control group (Figure 4D). An IP assay *in vitro* also showed that SGOL2 contributed to the stabilization of RAB1A by ubiquitination decrease (Figure 4D, 4E). Proteins that modified by ubiquitin are degraded generally through the proteasomal pathway. As the main pathway to mediate protein degradation, we tried to confirm whether SGOL2 delayed the degradation of RAB1A through inhibiting proteasome activities. Cell lines were both treated with proteasome inhibitor MG132. As shown in Figure 4F, SGOL2 mediated RAB1A stabilization partly in the proteasome-dependent pathway (Figure 4F). Thus, these results indicated that RAB1A expression regulated by SGOL2 was subject to inhibit proteasomal degradation.

### SGOL2 knockdown inhibited RAB1A expression to suppress prostate cancer progression

**Figure 5:**
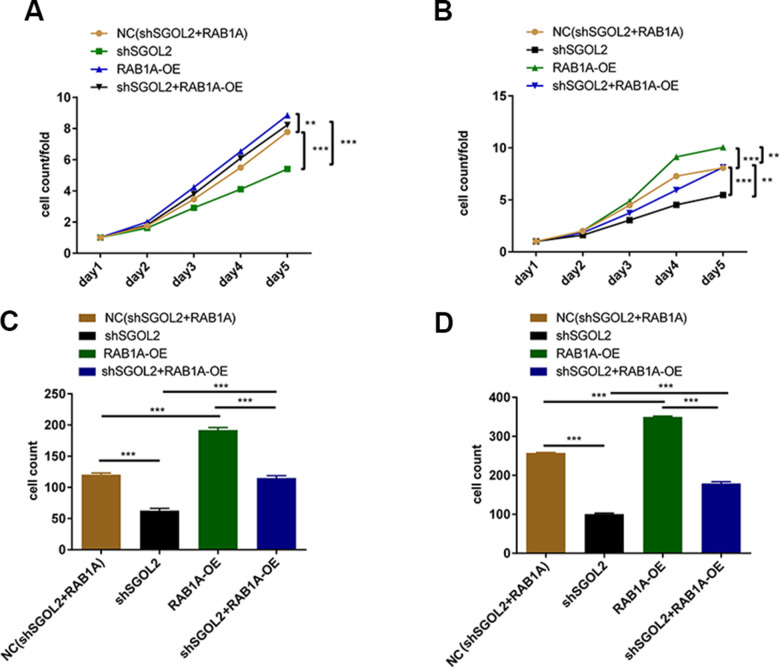
Control group, shSGOL2 + RAB1A-OE group, shSGOL2 group, RAB1A-OE group after transfecting with corresponding plasmids were subjected to the proliferation by Celigo cell counting assay in DU145 (**A**) and LNCaP (**B**), cell migration by transwell assay in DU145 (**C**) and LNCaP (**D**).

In order to confirm the role of SGOL2/RAB1A regulatory axis in prostate cancer, we constructed four cell models using SGOL2 knockdown lentivirus and RAB1A overexpression (OE) lentivirus (control group, shSGOL2 + RAB1A-OE group, shSGOL2 group, RAB1A-OE group). RAB1A overexpression promoted prostate cancer cell proliferation (Supplementary Figure 1E). The combination of shSGOL2 with RAB1A overexpression had similar inhibitory effects on tumor growth and reversed the shSGOL2 effects on proliferation ability (Figure 5A, 5B). Transwell migration assay revealed that RAB1A overexpression enhanced metastasis *in vitro* (Figure 5C, 5D). Taken together, SGOL2 was a novel regulator of RAB1A to regulate prostate cancer development *in vitro*.

### SGOL2 and RAB1A influenced TME of prostate cancer

**Figure 6:**
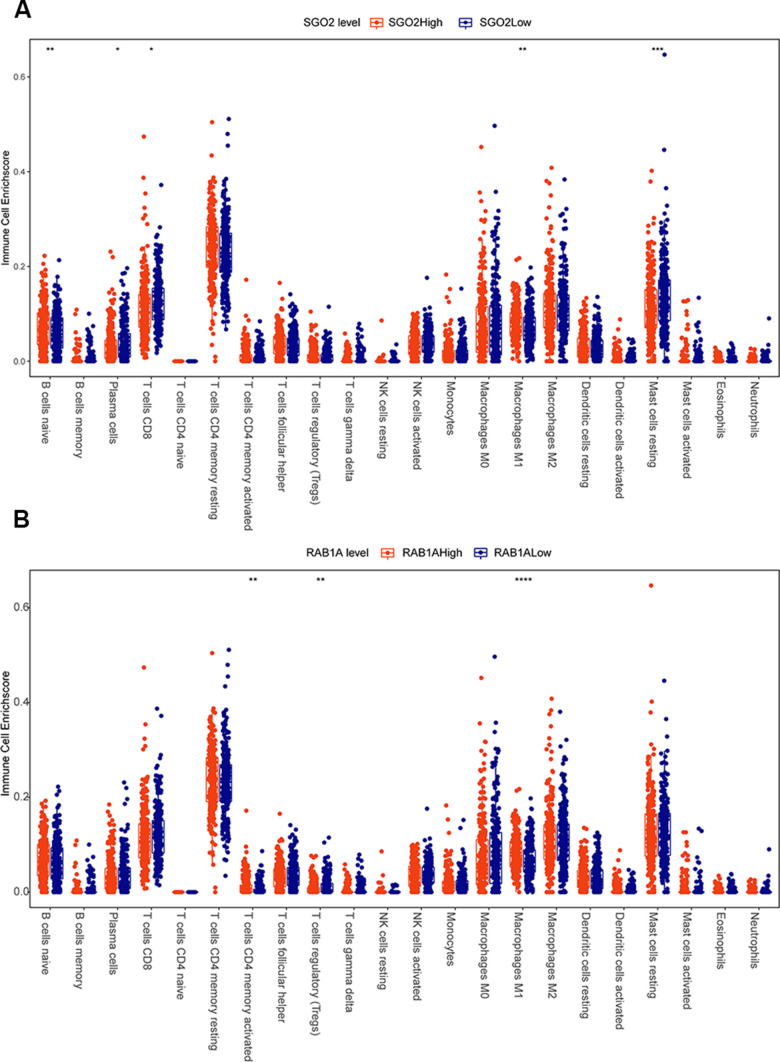
**SGOL2 and RAB1A may regulate the microenvironment of prostate cancer.** TCGA analysis displayed the relationship between SGOL2 (**A**), RAB1A (**B**) expression and immune cell enrichment.

To figure out whether SGOL2 and RAB1A influence TME in prostate cancer, we downloaded the dataset of prostate cancer patients (n=450) from the TCGA database. Correlational analysis of immune infiltration was calculated and visualized by corrplot package. Results showed that the immune microenvironment of the SGOL2-high group included more primary B cells and M1 macrophages, fewer CD8+ T cells, and resting mast cells compared to the SGOL2-low group (Figure 6A). Additionally, high RAB1A expression may induce activated CD4+T cells and M1 macrophages, in addition, inhibit Treg cells production (Figure 6B). Results presented that SGOL2 and RAB1A may regulate the TME in prostate cancer.

## DISCUSSION

Progression of prostate cancer is a complex multi-step process accompanied with genetic mutations and TME modulation [27]. In recent years, molecular characteristics of localized, metastatic, and recurrent prostate cancer patients have contributed to formulate therapeutic strategies. Given the current situation, an increasing body of studies focus on exploring underlying molecular mechanisms related to prostate cancer progression [28–30]. Herein, our findings showed that SGOL2, which expressed differently between prostate cancer tissue and para-cancer tissue, was positively associated with clinical stage, histopathological grade, lymphatic metastasis, and survival prognosis. *In vitro*, SGOL2 deletion inhibited proliferation and migration, increased cell apoptosis, along with the arrest of cell cycle S/G2. To explore the potential mechanism of how SGOL2 influenced tumor progression, we focused on the expression of its downstream proteins. Mass spectrometry, correlation analysis, and Co-IP jointly confirmed that SGOL2 played a critical role in RAB1A regulation in prostate cancer. Our findings revealed the oncogenic role of SGOL2 in the progression of prostate cancer, highlighting the tremendous possibility of SGOL2 being a promising biomarker for prostate cancer.

RAB GTPases, as part of the largest family of small GTPases, are involved in multiple diseases in many species, especially in humans [31]. RAB1A not only functions as a crucial regulator of vesicular transportation from endoplasmic reticulum to Golgi but also influences material metabolism [32, 33]. Moreover, RAB1A has been reported to activated mTORC1, Wnt, IL-4R/JAK1/STAT6 signaling pathways [34–36]. Additionally, a dynamic balance of ubiquitination and deubiquitination is a key mechanism controlling cellular RAB1A levels. For instance, USP2a, a deubiquitinating enzyme, stabilized RAB1A to promote cancer progression and metastasis in hepatocellular carcinoma [37]. E3 ubiquitin ligase RNF115 catalyzed RAB1A ubiquitination to interrupt TLRs transportation and TLRs-mediated immune response [38]. In the data reported herein, SGOL2 was for the first time found to inhibit the RAB1A ubiquitination process in a protease-dependent pathway. These reports, together with our findings, indicated that SGOL2 might play pivotal roles in prostate cancer development by upregulating RAB1A level.

Ubiquitin modification draws support from E1, E2, and E3 enzymes to complete the ubiquitination process and positively participates in cancer development. E3 ubiquitin ligases, as the most important components in the ubiquitination process, have the substrate specificity to target specific proteins [39]. However, there is no report revealing that SGOL2 acts as an E3 ubiquitin ligase or contains a specific domain or motif such as HECT, RING, and F-box [40–42]. Therefore, we conjectured that there existed one E3 ligase in the process of SGOL2-mediated RAB1A ubiquitination, which should be subsequently confirmed by a series of assays to figure out the involved E3 ubiquitin ligase. In this study, SGOL2 knockdown not only inhibited the degradation of RAB1A but also downregulated RAB1A mRNA expression (Supplementary Figure 1D). We confirmed that ubiquitination modification got involved in RAB1A regulation. As to transcriptional regulation, further studies are required to characterize this regulated manner.

Recent progress in immunotherapy based on checkpoint blockade has made no inspiring outcome in mCRPC. Chronic inflammation in the adult prostate is regarded as a risk factor contributing to tumorigenesis. Additionally, inflammation probably occurs in genomic mutations and rearrangements [43, 44]. RAB1A activated TLR4-dependent NF-KB signaling through transporting TLR4 to the membrane and promoted IL-1β and IL-18 generation and maturation [45]. To explore the underlying mechanism by which SGOL2 and RAB1A regulate TME in prostate cancer, SGOL2 and RAB1A both were predicted to regulate immune cell infiltration of prostate cancer, including lymphocytes and myeloid cells. Our findings indicated that SGOL2 probably stabilized RAB1A to regulate TME and this result contributed to our understanding of prostate cancer.

In summary, our study convincingly indicated that, as a new tumor promotor, the overexpression of SGOL2 was associated with increased proliferative and metastatic properties in prostate cancer. Further study revealed that SGOL2 inhibited RAB1A ubiquitination in a proteasome-dependent method. At last, we speculated both SGOL2 and RAB1A were involved in modulating TME, which provided a direction to develop the efficiency of prostate cancer treatment. Generally, our data demonstrated that SGOL2 and RAB1A might be used to detect cancer progression and act as potential therapeutic targets in prostate cancer.

## MATERIALS AND METHODS

### Immunohistochemistry (IHC) analysis

Tissue Microarray (HProA150CS01-M-018, OUTDO BIOTECH) was deparaffinized with xylene, rehydrated using ethanol and running water. Then 1×EDTA buffer was used for antigen retrieval at 100° C for 30 minutes and cooled to room temperature. TMA blocked with goat serum was incubated with SGOL2 antibody overnight at 4° C, then incubated with secondary anti-rabbit antibody. Incubated TMA was visualized by diaminobenzidine (AIDISHENG, ADS053W0) and counterstained with hematoxylin for 10-15 seconds. After washing with running water and dehydrating by ethanol, the coverslip was glued onto slides with neutral balsam. Immunoreactivity was scored by the percentage of the stained cells (0, no staining; 1, 0-25%; 2, 25-50%; 3, 50-75%; 4, ≥75%) and the intensity of staining in cellular plasma, membrane and nuclear (0, no color; 1, slight yellow; 2, yellow brown; 3, brown). Finally, multiply the two values and estimate the purpose of the TMA (0, negative; 1-4, positive +; 5-8, positive ++; 9-12, positive +++). According to the expression of SGOL2, we halved and analyzed the relationship between SGOL2 expression levels and clinical characteristics [46].

### Cell culture

Human prostate cancer cell lines 22RV1 and C4-2 were obtained from Bena Technology (Hangzhou, Zhejiang, China). DU145, PC-3, and LNCaP were respectively purchased from Research R&S (Shanghai, China), Genechem (Shanghai, China), and Fenghui Biotechnology (Changsha, Hunan, China). Normal prostate epithelial cell line WPMY was obtained from the Center for Excellence in Molecular Cell Science (Shanghai, China). 22RV1, PC-3, C4-2, LNCaP, and DU145 were cultured in RPMI 1640 (Viva Cell, C3010-0500) and 10% fetal bovine serum (FBS, Gibco,10091-148). RWPE-1 was cultured in Defined K-SFM (Gibco, 10744019) with 10% FBS. WPMY was cultured in DMEM (Meilunbio, MA0212) containing 10% FBS. All cell lines were cultured in a humidified incubator with 5% CO2 at 37° C.

### Plasmid construction and lentiviral packaging

RNA interference (RNAi) targeting SGOL2 was synthesized from Sangon Biotech (Shanghai, China) and overexpressed RAB1A was synthesized by BGI (Shanghai, China). Then, the combined plasmid, which was constructed by RNAi sequences and BR-V-108, pMD2.G vector plasmid, and pSPAX2 vector plasmid were transfected into 293T cells with Polyethylenimine, Linear, MW 25000, Transfection Grade (Polysciences, 23966-1) in Opti-MEM R1 (1×) (Gibco,1868811) for 6h and changed Opti-MEM R1 (1×) for DMEM (Gibco,41965062) with 10% FBS. The supernatant was gathered and spun to clear the cellular debris. The acquired samples were stored at -80° C [47].

### Primers

Primers in RT-qPCR: GAPDH, forward 5’-TGACTTCAACAGCGACACCCA-3’ and reverse 5’-CACCCTGTTGCTGTAGCCAAA-3’; SGOL2, forward 5’-TGAGATGAGAAACGCCCAGTC-3’ and reverse 5’-TTCCCAAGATGACCCACGCT-3’.

### RNA extraction and qRT-PCR

Total RNA was extracted from the cultured cells with TriQuick Reagent (Solarbio, R1100-500ml) following a standard protocol. The quality was assessed by Nanodrop 2000/2000C spectrophotometer (Thermo, Waltham, MA, USA). cDNA was acquired via reverse transcription on Hiscript QRT supermix for PCR (+gDNA WIPER) (Vazyme, R123-01) instruction manual. The relative quantitative analysis of gene expression was calculated by the 2^-ΔΔCt^ method compared to the control group.

### Western blotting (WB)

Total protein was extracted from the cultured cells with lysis buffer (Beyotime, P0013) and protein concentration was measured by BCA Protein Assay Kit (Beyotime, P0009). Protein was separated by 10% SDS-PAGE and transferred to the PVDF membranes. The membranes were blocked with TBST containing 5% no-fat milk for 1 hour and incubated with primary antibodies at 4° C overnight. After washing the membrane three times with 1×TBST, the membranes were incubated with specified secondary antibodies for 1 hour. Then these membranes were illuminated by using an immobilon Western Chemiluminescent HRP Substrate kit (Millipore) after washing with 1×TBST [46]. All the samples were washed twice in phosphate-buffered saline (PBS) before isolating the proteins.

### Co-immunoprecipitation (Co-IP) and IP assay

Cultured cells were lysed in lysis buffer (Beyotime, P0013) with a protease inhibitor and the concentration of protein was measured by BCA Protein Assay Kit (Beyotime, P0009). For Co-IP, SGOL2 and RABIA antibodies mixed with magnetic beads were incubated with protein samples overnight at 4° C. Separate proteins from magnetic beads and detect protein by WB as mentioned above. For IP, a ubiquitin antibody was used to detect the ubiquitination of proteins. Antibodies were arranged in Supplementary Table 1.

### Mass spectrometry (MS)

Cells with SGOL2 deletion and controlled cells were lysed. Then proteins were extracted from cells and measured concentration. 10% SDS-PAGE were stained with Coomassie Blue Staining Solution for 1 hour or more. After Stained SDS-PAGE was decolorized, selected appropriate parts to detect protein via MS technology.

### CCK8 assay, wound-healing assay, transwell assay

The viability of cells was evaluated by a CCK8 assay following the manufacturer’s instructions. Cells infected for 48h were trypsinized and seeded 3000 cells per well in a 96-well plate. After that, cells were cultured in 100ul complete medium and added 10ul CCK8 (Meilunbio, MA0218) per well. After incubating for 3 hours at 37° C, the absorbance at 450nm was detected at 24h, 48h, 72h, 96h and 120h.

Wound-healing assay was used to detect migratory ability. Cells were seeded in a 96-well plate of 50000 cells/well and cultured in 100ul complete medium. Wound healing assay was prepared by drawing a vertical line in the center of the well, changing the medium with no FBS, and detecting the weight of lines to assess the migratory ability.

Transwell assay was performed via Boyden chamber with 8.0μm pore size (Corning, CLS3422) where 50000 cells were incubated at 37° C. Add 100ul RPMI 1640 in the upper chamber and 600ul RPMI 1640 with 30% FBS in the lower chamber. After 20 hours of incubation at 37° C with 5% CO2, metastatic cells were dyed with 0.1% Crystal Violet Stain solution (Solarbio, G1063) for 3 min.

### Apoptosis assay and cell cycle assay

Cells were trypsinized and washed with Annexin V Binding Buffer (10 ×, diluted with PBS) (Invitrogen, 2094083). And add 10ul Annexin V-APC (Elascience, E-CK-A117) in a concentration of 7-8×105 cells/ml, then incubated for 15 minutes in the dark to detect the percentage of apoptosis. Cell cycle assay was detected on the manufacturer’s instructions.

### Celigo cell counting assay

Cells infected for 48h were trypsinized and seeded 3000 cells per well in a 96-well plate. After that, cells were cultured in 100ul complete medium at 37° C with 5% CO2 for 5 days. Celigo image cytometer (Nexcelon Bioscience, Lawrence, MA, USA) counted cell number every day.

### Ubiquitination assays

Cells infected by lentivirus were cultured in 6-well plates or 10 cm plates for 5 days. DU145 and LNCaP cells were treated with 10mmol/ml cycloheximide (CHX) (Selleckchem, S741802) in 1.5ml complete medium at several time points. Proteasome inhibitor MG132 (10mmol/ml) (Mechem Express, HY-13259) was mixed with 1.5ml complete medium per cell in 6-well plates and 7.5ml complete medium in 10cm dishes for 8h. Cells were respectively subjected to WB and IP.

### Samples and data preprocessing

A total of 450 cases of prostate cancer were downloaded from The Cancer Genome Atlas (TCGA, https://portal.gdc.cancer.gov/) and further assessed the infiltration of the immune cell using the Cibersort algorithm and ssGSEA algorithm of the GSVA package [48]. The expression data of 450 samples was divided into SGOL2-high group and SGOL2-low group based on median expression of SGOL2, and so did RAB1A. Correlation analysis of immune infiltration was calculated and visualized by corrplot package. Data analysis mentioned above was processed via R 4.0.3.

### Statistical analysis

Continuous variables were shown as the mean ± SD, P values, and standard deviation using Student’s t-test, in which P<0.05 was considered statistically significant, and one-way ANOVA was used to compare multiple separate groups. All statistical analysis was performed using SPSS 17.0 (IBM, SPSS, Chicago, IL, USA) and Graphpad Prism 6.01 (Graphpad Software, La Jolla, CA, USA). Apply Mann-Whitney U analysis and Spearman analysis to evaluate the correlation between the two groups. *, P < 0.05; **, P < 0.01; and ***, P < 0.001.

## Supplementary Material

Supplementary Figure 1

Supplementary Tables
