# Supplementary material for: SGOL2 promotes prostate cancer progression by inhibiting RAB1A ubiquitination
Source: Aging (Albany NY). 2022 Dec 23;14(24):10050–66. doi: 10.18632/aging.204443 (PMC9831743; doi:10.18632/aging.204443)
Supplement: Supplementary Figure 1 [file aging-14-204443-s001.pdf]

SUPPLEMENTARY FIGURE

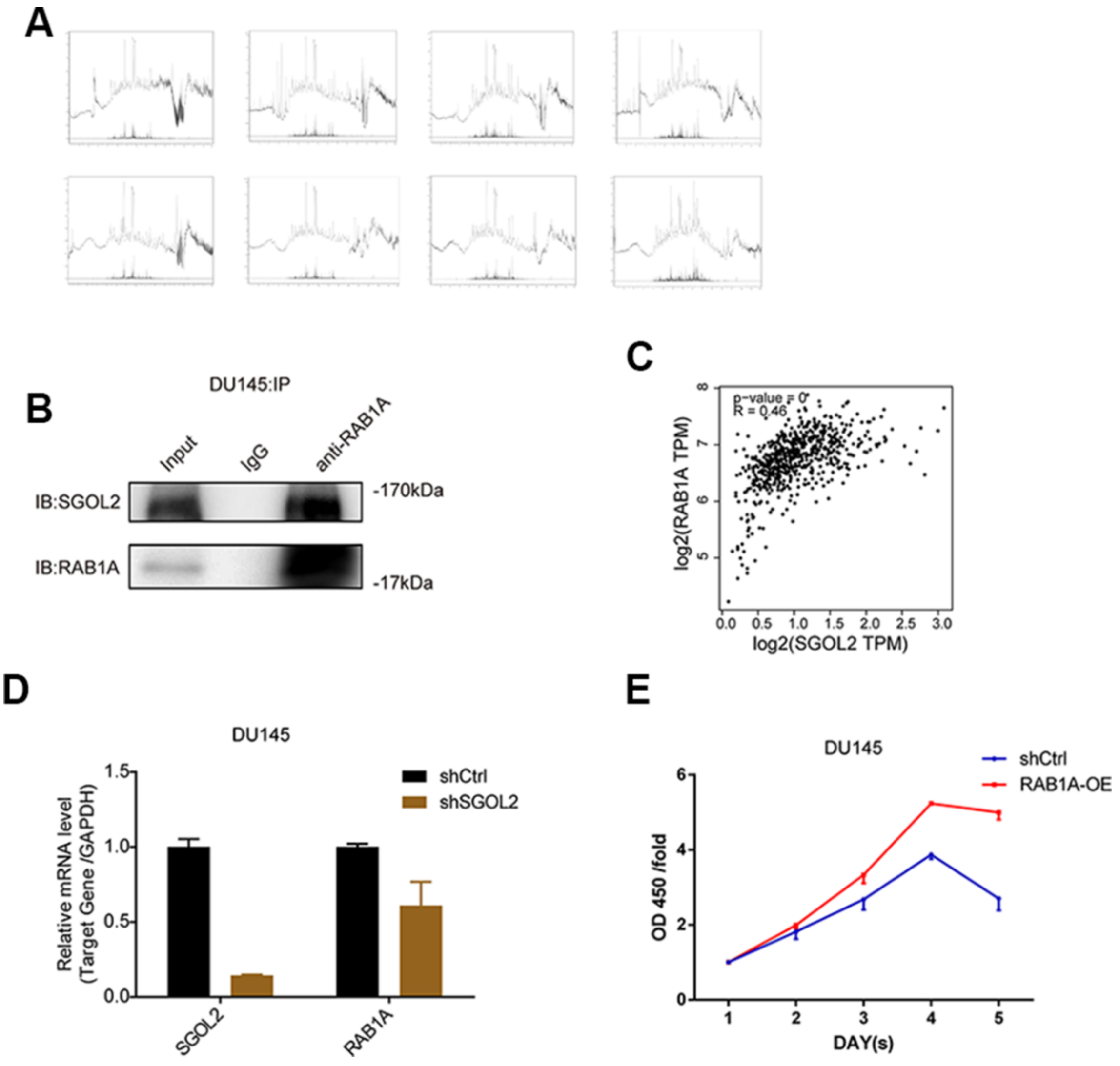

Supplementary Figure 1. (**A**) 8 samples were cut from SDS-PAGE mentioned in Figure 4A. (**B**) WB analysis for Co-IP detected the interaction between RAB1A and SGOL2 in DU145. (**C**) GEPIA 2.0 presented the image of correlative analysis between SGOL2 and RAB1A. (**D**) The level of RAB1A mRNA was detected in shSGOL2 and the control group. (**E**) Growth curves of CCK8 assay were detected in RAB1A overexpressed group and the control group in DU145.
