# Supplementary material for: SGOL2 promotes prostate cancer progression by inhibiting RAB1A ubiquitination
Source: Aging (Albany NY). 2022 Dec 23;14(24):10050–66. doi: 10.18632/aging.204443 (PMC9831743; doi:10.18632/aging.204443)
Supplement: Supplementary Tables [file aging-14-204443-s002.pdf]

SUPPLEMENTARY TABLES

Supplementary Table 1. Antibodies used in WB, Co-IP, and IHC.

Primary antibodies	Dilution in WB	Source species	Company	Catalog No.
SGOL2	1:1000	Rabbit	Bioss	bs-21166R
RAB1A	1:1000	Rabbit	Proteintech	11671-1-AP
Ubiquitin	1:1000	Mouse	CST	3936S
GADPH	1:3000	Mouse	Proteintech	60004-1-lg
**Primary antibodies**	**Dilution in Co-IP**	**Source species**	**Company**	**Catalog No.**
SGOL2	1:50	Rabbit	Novus	NB100-60455
RAB1A	1:100	Rabbit	Proteintech	11671-1-AP
**Primary antibodies**	**Dilution in IHC**	**Source species**	**Company**	**Catalog No.**
SGOL2	1:100	Rabbit	Abcam	ab122258
**Secondary antibodies**		**Dilution**	**Company**	**Catalog No.**
HRP Goat Anti-Rabbit IgG (WB)		1:3000	Beyotime	A0208
HRP Goat Anti-Mouse IgG (WB)		1:3000	Beyotime	A0216
Goat Anti-Rabbit IgG H&L (HRP)		1:400	Abcam	ab97080
IgG (IHC)			Beyotime	A7016

Supplementary Table 2. Relationship between SGOL2 expression and tumor characteristics in patients with prostate cancer.

		SGOL2
Gleason Score	Spearman correlation	0.213*
	(two-tailed)	0.042*
	N	91
Grade	Spearman correlation	0.285*
	(two-tailed)	0.006**
	N	91
lymphatic metastasis	Spearman correlation	0.341**
	(two-tailed)	0.001**
	N	91
Stage	Spearman correlation	0.409**
	(two-tailed)	0.000***
	N	91

P<0.05; **, P<0.01; ***, P<0.001.
